# A new species of *Pardalisca* Krøyer, 1842 (Crustacea, Amphipoda, Pardaliscidae) from the Clarion-Clipperton Zone in the abyssal central east Pacific

**DOI:** 10.3897/zookeys.1274.140692

**Published:** 2026-03-24

**Authors:** Karolina Biniek, Ed A. Hendrycks, Anna M. Jażdżewska

**Affiliations:** 1 Department of Invertebrate Zoology and Hydrobiology, Faculty of Biology and Environmental Protection, University of Lodz, Banacha 12/16, 90-237 Lodz, Poland Canadian Museum of Nature, Research Associate, Research and Collections Ottawa Canada; 2 Canadian Museum of Nature, Research Associate, Research and Collections, P.O. Box 3443, Station D, Ottawa, K1P 6P4, Canada University of Lodz Łódź Poland

**Keywords:** Amphipods, deep-sea mining, depth record, DNA barcode

## Abstract

The Pardaliscidae is one of the most abundant and species-rich families of Amphipoda in the deep sea. Among pardaliscid species collected from abyssal depths (4185–4182 m) of the Clarion-Clipperton Zone as part of the MANGAN 2023 cruise, a single large representative of the genus *Pardalisca* was recovered. The species was confirmed as new to science and is here illustrated and described in detail; a confocal laser scanning microscope (CLSM) image and a molecular barcode are also provided. *Pardalisca magdalenae***sp. nov**. differs from its congeners by the shape of the dorsal process on urosomite 1 as well as the morphology of the gnathopod dactyls.

## Introduction

Amphipods of the family Pardaliscidae Boeck, 1871 are recognized as a diverse and abundant component of deep-sea communities. These predatory amphipods are known to be good swimmers and are considered epibenthic or demersal (Hendrycks and Conlan 2003; Brix et al. 2018). The family consists of 23 genera and 87 species (Horton et al. 2024). The genus *Pardalisca* Krøyer, 1842 is the third most species-rich within the family and presently consists of 11 species (Horton et al. 2024). Karaman (1974) revised the family Pardaliscidae, and since that time there has been no overview or revision of the genus *Pardalisca*. However, Biswas et al. (2009) have provided a detailed key to Pardaliscidae. *Pardalisca endeavouri* Shaw, 1989 is the most recently described species and the first described from geothermal vents.

*Pardalisca* species are characterised by the following combination of characters: antenna 1 shorter than antenna 2, peduncles short; lateral cephalic lobe broadly rounded; upper lip asymmetric; maxilla 1 palp article 2 very widened; maxilla 2 lobes narrowed; maxilliped with small inner lobes, outer lobes well developed, medially strongly elongated and concave; mandibles asymmetric with short 3-articulate palp; gnathopods 1–2 simple, carpus dominant with dactyls usually toothed; pereopods 3–7 simple; urosome 1–2 with dorsal ornamentation and telson long and deeply cleft. Morphological study of the Clarion-Clipperton Zone (CCZ) specimen clearly determined that it belonged to the genus *Pardalisca* and further, that it is a species new to science.

Here we add a new species to the genus *Pardalisca* collected from the CCZ, the area prospected for deep-sea mining, from a depth of 4185–4182 m, which is a depth record for the genus.

## Material and methods

### Specific methodology description

The single specimen in the present study was sampled in the central east Pacific, specifically within the easternmost sector of the Clarion-Clipperton Zone (**CCZ**). The specimen was collected with an epibenthic sledge (**EBS**) during the MANGAN 2023 cruise (Rühlemann et al. 2023). For details of gear deployment and sample processing, see Jażdżewska and Horton (2026) and Jażdżewska et al. (2025).

The specimen was initially examined using a Leica M125 and a Nikon SMZ800 stereomicroscope. The habitus of the holotype is presented as a photograph obtained with a confocal laser scanning microscope (**CLSM**). The holotype was stained in Congo red and acid fuchsin, temporarily mounted onto slides with glycerin and examined with a Leica TCS SPV equipped with a Leica DM5000 B upright microscope and three visible-light lasers (DPSS 10 mW 561 nm; HeNe 10 mW 633 nm; Ar 100 mW 458, 476, 488 and 514 nm), combined with the LAS AF 2.2.1 software (Leica Application Suite, Advanced Fluorescence). A series of photographic stacks were obtained, collecting overlapping optical sections throughout the whole preparation (Michels and Büntzow 2010; Kamanli et al. 2017).

Afterwards, the specimen was dissected and mounted on permanent slides using Euparal (Waldeck GmbH & Co.). Appendages on slides were examined using a Nikon Eclipse Ci compound microscope equipped with a camera lucida. Pencil drawings from the microscope were used as the basis for line drawings. The drawings were inked with Adobe Illustrator ® following the recommendations of Coleman (2003, 2009).

In the descriptions and figures the following abbreviations were used: **A1, 2** = antenna 1, 2; **G1, 2** = gnathopod 1, 2; **LL** = lower lip; **Md** = mandible; **Mx1, 2** = maxilla 1, 2; **Mxp** = maxilliped; **P3–7** = pereopod 3–7; **T** = telson; **U1–3** = uropod 1–3; **UL** = upper lip; **l** = left; **r** = right.

The individual was subjected to cytochrome *c* oxidase subunit I gene (COI) barcoding prior to identification of the species. The molecular procedures are described in Jażdżewska et al. (2025). The sequence was deposited in Barcode of Life Data Systems (BOLD, Ratnasingham and Hebert 2007) and in GenBank with the accession number: PQ734367. The relevant voucher information, taxonomic classification and sequence are deposited in the data set “DS-AMPHICCZ” in BOLD (https://doi.org/10.5883/DS-AMPHICCZ) (http://www.boldsystems.org).

## Results

### Systematics

**Order Amphipoda Latreille, 1816**

**Suborder Amphilochidea Boeck, 1871**

**Superfamily Dexaminoidea Leach, 1814**

**Family Pardaliscidae Boeck, 1871**

#### Genus Pardalisca

Taxon classificationAnimaliaAmphipodaPardaliscidae

Krøyer, 1842

95EB0D6C-60E8-5B7C-B32C-C1C3D9A63EDF

Pardalisca Krøyer, 1842: 153.

##### Type species.

*Pardalisca cuspidata* Krøyer, 1842 (type by monotypy).

##### Included species.

*Pardalisca* contains 12 species: *Pardalisca abyssi* Boeck, 1871; *P. abyssoides* K.H. Barnard, 1932; *P. australiensis* K.H. Barnard, 1931; *P. brachydactyla* Bellan-Santini, 1985; *P. cuspidata* Krøyer, 1842; *P. endeavouri* Shaw, 1989; *P. magdalenae* sp. nov.; *P. magellanica* Schellenberg, 1931; *P. marionis* Stebbing, 1888; *P. mediterranea* Bellan-Santini, 1985; *P. tenuipes* G.O. Sars, 1893; *P.* sp. J.L. Barnard, 1967.

#### Pardalisca magdalenae sp. nov.

Taxon classificationAnimaliaAmphipodaPardaliscidae

7D453727-025D-5D47-A8E3-42D54F7D7D5C

https://zoobank.org/7FD9C24A-7C3A-4683-8317-1942ACA2319E

Figs 1, 2, 3, 4, 5

##### Type material.

***Holotype***: Pacific Ocean • male, 17 mm, Clarion-Clipperton Zone; BGR exploration contract area, R/V *Kilo Moana*, MANGAN 2023, EBS, KM23-50, 01 May 2023, 11°17.79'N, 116°18.86'W–11°18.54'N, 116°17.67'W, 4185–4182 m, SMF 63360, COI: PQ734367.

##### Type locality.

Abyssal Pacific Ocean, Clarion-Clipperton Zone, 11°17.79'N, 116°18.86'W–11°18.54'N, 116°17.67'W, 4185–4182 m.

##### Etymology.

This species is named for Magdalena Biniek, the sister of the senior author.

##### Diagnosis.

Lateral cephalic lobe with very small, subacute process between antennae 1–2, broadly rounded ventrally; gnathopods 1–2 simple with massive carpus; dactyls long, acute, length 0.6 × propodus, with an anterodorsal midmarginal tooth and 5 medial teeth at midpoint of its length; urosome 1 with large, rounded dorsal process directed posteriorly; urosome 2 with a single dorsal tooth directed posteriorly; urosome 3 with low dorsal boss; maxilla 1, palp article 2 strongly dilated, nearly circular. Maxilla 2, plates strongly narrowed. Telson cleft 90%, lobes not narrowed.

##### Description.

Based on holotype male, 17 mm, registration number SMF 63360.

**Figure 1.**
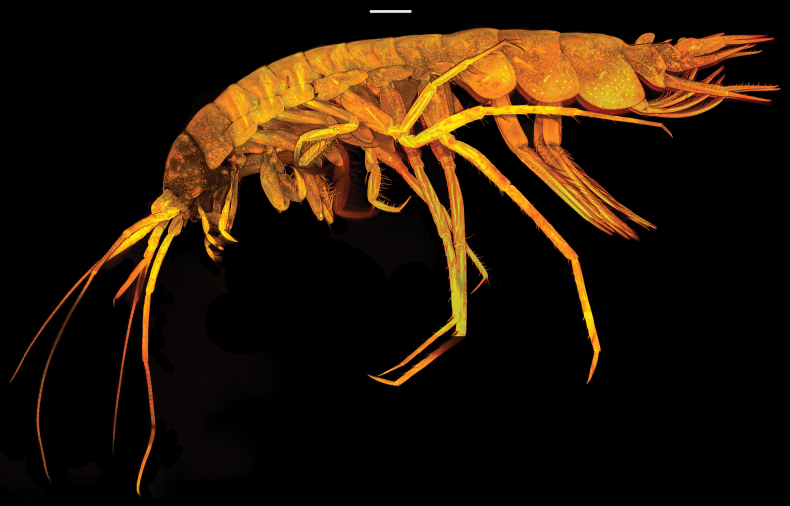
*Pardalisca magdalenae* sp. nov. Habitus, CLSM photography, holotype male, 17 mm, SMF 63360. Scale bar: 1 mm

**Figure 2.**
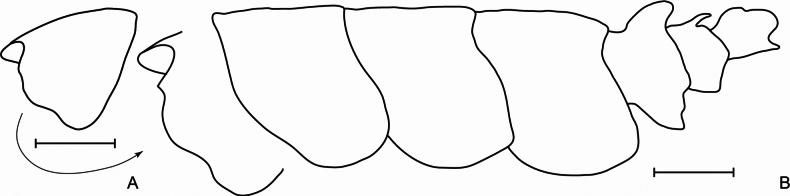
*Pardalisca magdalenae* sp. nov. holotype male, 17 mm, SMF 63360. **A** Head profile **B** Pleonites and urosomites profile. Scale bars: 1 mm.

***Body*** (Figs 1, 2): slender, pereonites 1–7 and pleonites 1–3 smooth. ***Urosomite 1*** (Figs 1, 2): dorsally with a large, upright rounded carination (boss) directed slightly posteriorly and a lateral ridge. ***Urosomite 2*** (Figs 1, 2): with an acute tooth directed posteriorly. ***Urosomite 3*** (Figs 1, 2): with a low, rounded dorsal boss. ***Epimeron 1*** (Figs 1, 2): ventral margin with 3 small spines, posteroventral corner rounded. ***Epimeron 2*** (Figs 1, 2): quadrate, ventral margin convex, with 3–4 small spines, posteroventral corner rounded. ***Epimeron 3*** (Figs 1, 2): similar in shape to epimeron 2 but larger, ventral margin convex with 4–5 small spines, posteroventral corner not produced, rounded, posterior margin slightly convex. ***Coxae 1*–*7*** (Fig. 1): shallow, subrectangular, much shorter than corresponding pereonites.

***Head*** (Figs 1, 2): about as deep, as long, slightly shorter than pereonites 1–2 combined; rostrum short, rounded, reaching 0.25 × length of the article 1 of antenna 1.

***Lateral cephalic lobe*** (Figs 1, 2): with a very small, subacute process between antennae 1–2, broadly rounded ventrally, with a very shallow sinus.

***Eye*** (Fig. 1): no eyes or ocular pigment visible in the preserved specimen.

**Figure 3.**
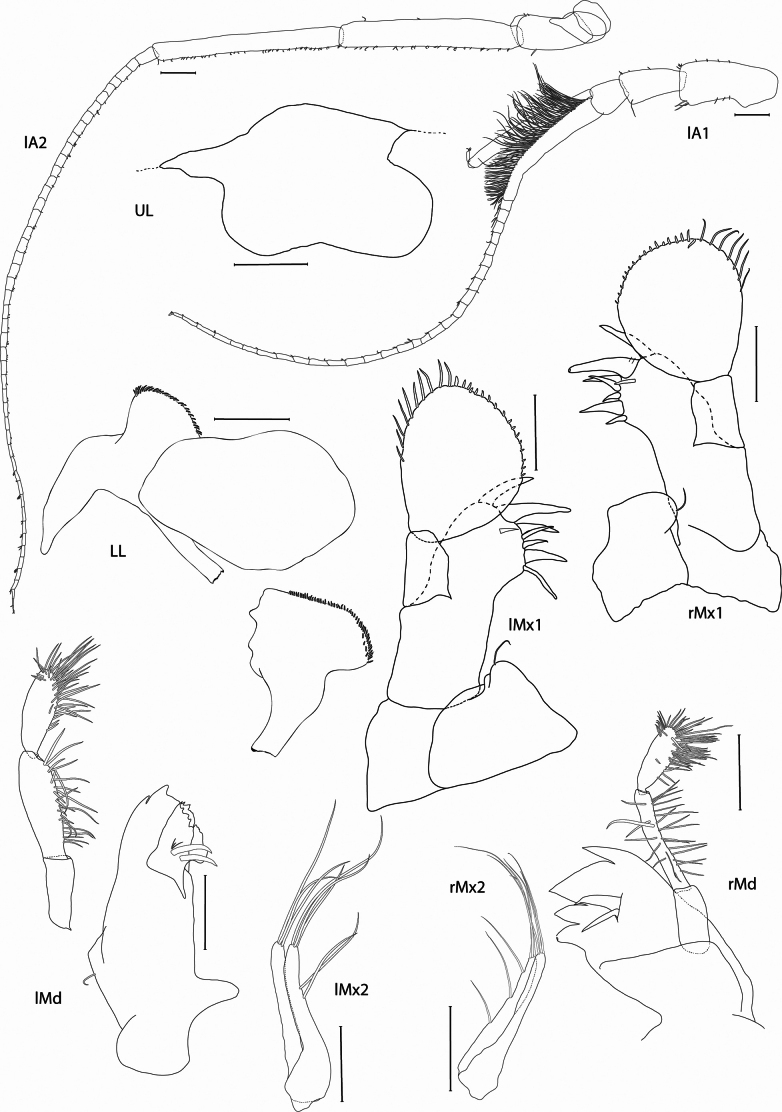
*Pardalisca magdalenae* sp. nov. holotype male, 17 mm, SMF 63360. Scale bars: 0.3 mm.

***Antenna 1*** (Figs 1, 3): long, length about 0.4 × body, length ratio of peduncular articles 1–3 1:0.6:0.4; proximolateral margin of peduncle 1 with a distinct protuberance; peduncle 2 widening distally; primary flagellum 31-articulate, with sparse setae placed distally on flagellar articles, first article furnished with strong callynophore, slightly shorter than peduncular articles 1–2 combined, densely furnished medially with aesthetascs, accessory flagellum well developed, 2-articulate, first article blade-like and similar in length to the callynophore, second article minute and furnished with group of setae.

***Antenna 2*** (Figs 1, 3): long, length about 0.5 × body, and 1.3 × antenna 1; peduncular article 1 small and round, article 2 with blunt gland cone reaching half-length of peduncle article 3, article 3 shorter than article 4, relative lengths of peduncle articles 3–5 1:2.9:3, articles 4–5 with many short brush setae on the inner edge; flagellum 46-articulate, with sparse setae distally on flagellar articles.

***Upper lip*** (Fig. 3): lobes asymmetric, anteroventrally emarginate.

***Mandible*** (Fig. 3): left incisor margin irregularly dentate; left lacinia mobilis finely serrate; accessory spine row with 2 spines and 3 fine setules; palp 3-articulate, slightly shorter than mandible body, articles 1–3 in ratio of 1:1.6:1.2, article 2 with 22 setae on inner margin, article 3 rounded and widening distally, inner margin and distally strongly setose, with about 35 setae; right incisor margin with 4 teeth, the second much larger than the others; right lacinia mobilis absent; accessory spine row with 2 spines, right mandibular palp twisted (not described).

***Lower lip*** (Fig. 3): damaged, outer lobes covered with fine setae, mandibular lobes narrow; inner lobes fused.

***Maxilla 1*** (Fig. 3): left and right similar; inner plate small, rounded with 1 distal seta; outer plate with 8 acute setal-teeth, second the strongest (7 apical/marginal larger and 1 subapical/submarginal smaller tooth); palp 2-articulate, article 1 0.5 × length of article 2, slender; article 2 strongly dilated, nearly circular, with 7–10 long apicolateral setae and 17 small spines apicomedially.

***Maxilla 2*** (Fig. 3): left and right similar; outer and inner plates slender, subequal, both with 3 long setae apically; inner plate wider proximally; inner plate of left maxilla 2 with 2 long setae on inner margin (in the same insertion position); inner plate of right maxilla 2 with 3 long separated setae on inner margin.

**Figure 4.**
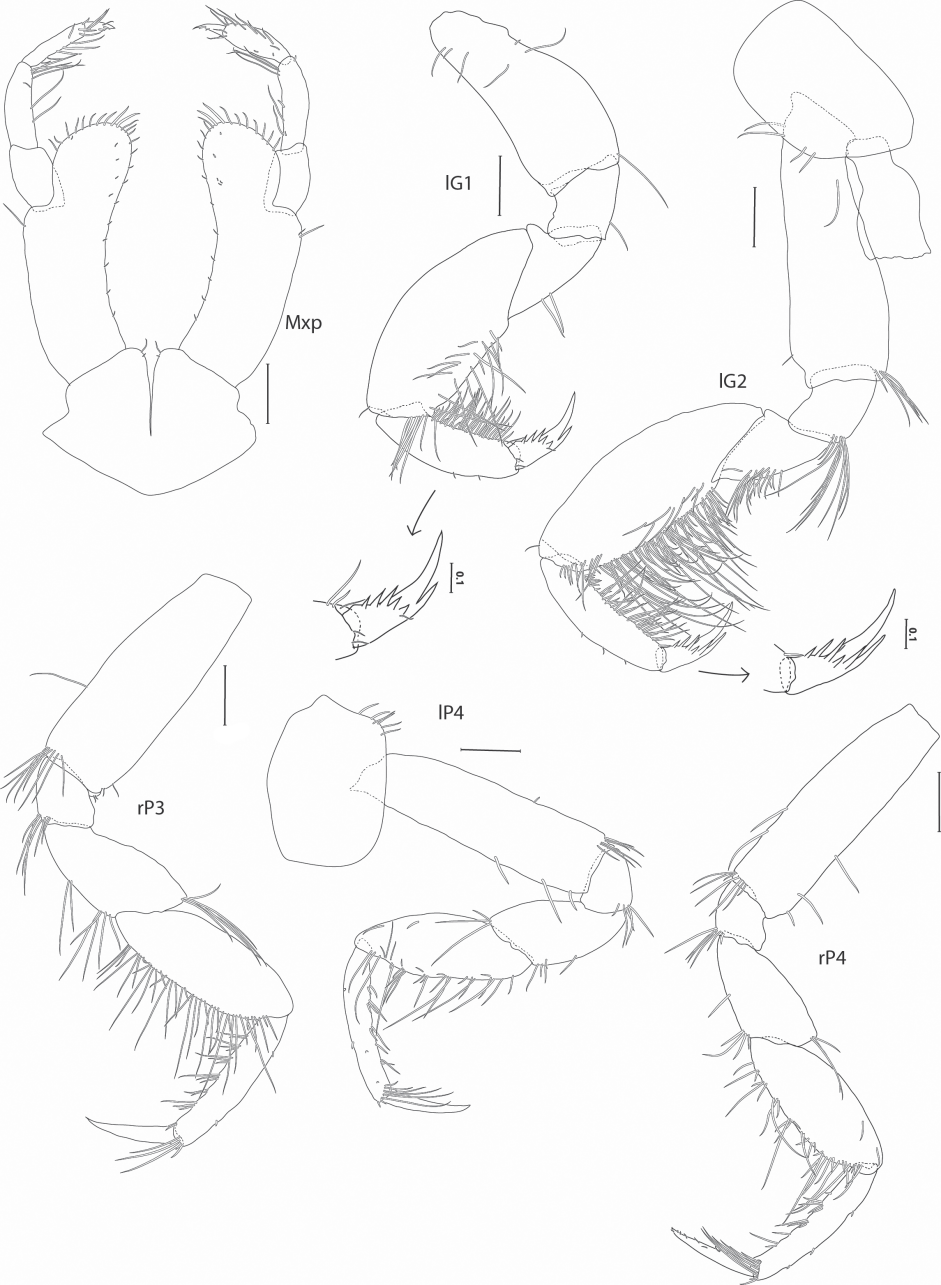
*Pardalisca magdalenae* sp. nov. holotype male, 17 mm, SMF 63360. Scale bars: 0.3 mm, unless indicated otherwise.

***Maxilliped*** (Fig. 4): inner plate very small, rounded, with 2 tiny setae apicomedially; basal section of outer plate lateral margin elongated and slender, length of the outer plate is 0.8 × the length of the palp, inner margin strongly concave, with about 8 equally spaced small setae, outer plate rounded, with about 13 apical setae; palp slender, article 2 longest, article 4 short, length 0.4 × article 3.

***Gnathopod 1*** (Figs 1, 4): simple; coxa subrectangular, length 1.6 × width, anterior margin slightly convex, anteroventral corner rounded; basis robust, length 2.1 × width, expanded distally; ischium subrectangular, posteroventral corner with a seta; merus triangular, posterior margin with 2 setae; carpus massive, subovate, width about equal to basis, length 2.4 × width, with 2 rows of setae; propodus length 0.6 × carpus, narrower, posterior margin straight, strongly setose, setae arranged in 2 rows; dactylus long, length 0.6 × propodus, with an anterodorsal midmarginal tooth and 5 medial teeth in the middle of its length.

***Gnathopod 2*** (Figs 1, 4): simple; coxa subrectangular, length 1.6 × width, anterior and ventral margins straight; basis narrower than gnathopod 1, length 3 × width, posterodistal margin with cluster of setae; ischium quadrate, posterodistal margin with cluster of setae; merus triangular, posterior margin with setae; carpus large, but smaller than gnathopod 1, length 2.8 × width, posterior margin straight, strongly setose; propodus much narrower than carpus, length 4.3 × width, strongly setose with setae of different lengths; dactylus long, length 0.6 × propodus, with an anterodorsal midmarginal tooth and 5 medial teeth in the middle of its length.

***Pereopod 3*** (Figs 1, 4): coxa subrectangular, length 1.8 × width, posteroventral corner with 2–3 short setae; basis slightly expanded distally, length 3 × width, posterior margin with a single seta, posterodistal corner with 8 long setae; ischium subrectangular, posterodistal corner with 6 long setae; merus widening distally, shorter than carpus, anterodistal corner and posterior margin with 1 and 3 clusters of long setae, respectively; carpus suboval, length 1.3 × merus, posterior margin strongly setose; propodus narrower than carpus (0.5 ×), length 0.9 × carpus, posterior margin with 6 groups of setae, and fringe of long setae at distal corner; dactylus long and slender, length 0.6 × propodus.

***Pereopod 4*** (Figs 1, 4): coxa subrectangular, similar to coxa 3 but with 6 setae; rest of pereopod similar to pereopod 3 except posterior margin of carpus weakly setose and fringe of setae near dactylus more numerous.

**Figure 5.**
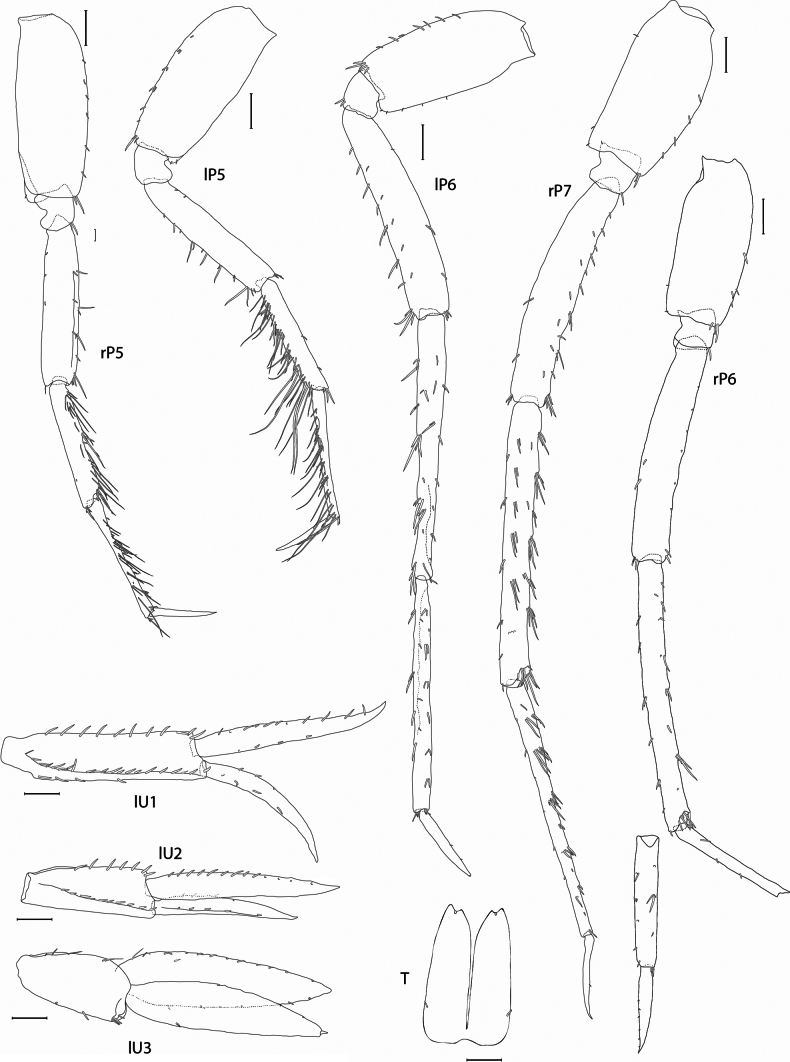
*Pardalisca magdalenae* sp. nov. holotype male, 17 mm, SMF 63360. Scale bars: 0.3 mm.

***Pereopod 5*** (Figs 1, 5): coxa weakly bilobed, anterior margin broadly rounded, posterior margin narrowly rounded, ventral margin slightly incised; basis narrowly rectangular, length 2.5 × width, anterior margin with 6 groups of short setae, posterior margin straight; merus slightly (1.2 ×) longer than carpus, with groups of setae along anterior margin; carpus long and slender, 0.8 × length of merus, anterior margin strongly setose; propodus slender, similar in length to carpus, anterior margin with groups of setae; dactylus slender, length 0.5 × propodus.

***Pereopod 6*** (Fig. 5): similar to pereopod 5 except merus, carpus and propodus relatively longer and anterior margins weakly setose, dactylus slightly shorter, length 0.4 × propodus.

***Pereopod 7*** (Fig. 5): similar in proportions to pereopod 6 except merus, carpus and propodus slightly longer.

***Pleopods 1*–*3*** (Fig. 1): robust, both peduncles and rami large, rami long and strongly setose.

***Uropod 1*** (Fig. 5): lanceolate; peduncle length equal to inner ramus, proximofacial, dorsolateral and dorsomedial margins with 4–5, 21 and 11 spines, respectively; inner ramus dorsolateral and dorsomedial margins with 4 and 11 spines respectively; outer ramus length 0.8 × inner ramus, dorsolateral and dorsomedial margins with 4 and 3 small spines, respectively.

***Uropod 2*** (Fig. 5): lanceolate; shorter than uropod 1; peduncle length 0.7 × inner ramus, dorsolateral and dorsomedial margins with 10 and 7 spines, respectively; inner ramus dorsomedial margin with 13 spines; outer ramus length 0.75 × inner ramus, dorsolateral and dorsomedial margins with 5 and 4 small spines, respectively.

***Uropod 3*** (Fig. 5): peduncle length 0.5 × inner ramus, dorsolateral and dorsomedial margins with 4 and 5 setae and spines, respectively; rami broadened, of similar size, outer ramus with a tiny article at distal end, dorsolateral and dorsomedial margins with 4 and 6 setae and spines, respectively (some plumose setae may be broken off); inner ramus, dorsolateral and dorsomedial margins with 6 and 13 setae and spines, respectively.

***Telson*** (Fig. 5): length 1.6 × width, cleft 90%, lobes not narrowed, distally with a small notch (spine in notch missing), proximodorsal margin with 1 seta on each lobe.

##### Intraspecific variation.

Unique, no other specimens to compare.

##### Molecular identification.

Following the definition given by Pleijel et al. (2008), the sequence of the holotype male of *P. magdalenae* sp. nov. (SMF 63360, GenBank accession number PQ734367) is designated as a hologenophore of all obtained sequences. The species has also received a Barcode Index Number from Barcode of Life Data Systems: BOLD:AFU4686 (https://doi.org/10.5883/BOLD:AFU4686).

##### Remarks.

The genus *Pardalisca* encompasses a rather homogeneous group of pardaliscid species which show a similar body shape, especially in regard to the head shape with broadly rounded lateral cephalic lobe, antenna 1 longer than antenna 2, gnathopods simple with dominant carpus and toothed dactyls, pereopods basic, simple, pleon powerful with robust pleopods, uropods lanceolate, spinose, telson cleft, and urosome 1–2 with dorsal ornamentation. With reference to the urosome ornamentation, species normally have bidentate teeth or cusps on the posterodorsal margin of urosomite 1, followed by a single cusp or tooth on urosomite 2. Urosomite 3 is normally smooth, lacking any process. The new species *P. magdalenae* sp. nov. differs from all other *Pardalisca* species in the possession of a large, rounded posterodorsal process on urosomite 1 instead of bidentate teeth or spines. Additionally, urosomite 3 has a low rounded dorsal boss (vs dorsally smooth in all other species). The morphology of the gnathopod dactyls of *P. magdalenae* sp. nov. are unlike any other *Pardalisca* species. Generally, the gnathopod dactyls of *Pardalisca* species are furnished with spines haphazardly arranged (with an indistinct pattern) or on the posterior margin with no discernible pattern (e.g. see Bellan-Santini 1985: fig. 7 for *P. mediterranea*; Shaw 1989: fig. 1 for *P. endeavouri*). In *P. magdalenae* sp. nov. the spination of both gnathopods 1–2 dactyls is very similar and in a distinct pattern, with a single, anterodorsal midmarginal tooth and 5 medial teeth running across the middle of the dactyls. These characters easily separate our new species from all other *Pardalisca* species.

Karaman (1974) gave citations pertaining to four species which had Pacific Ocean records: *P. australiensis* from Outside Trinity Opening, South Pacific near the Great Barrier Reef; *P. cuspidata* from NW Pacific, Kuril Islands (Gurjanova 1964); *P tenuipes* from the Eastern Pacific off southern California (Barnard 1962), and *Pardalisca* sp. J.L. Barnard, 1967 from Cedros Trench, off Baja California. Subsequently, *P. endeavouri* was described from an area of geothermal vents (Explorer Ridge) off the west coast of Vancouver Island. The records of both *P. cuspidata* and *P. tenuipes*, from the Pacific may be misidentifications or cases of cryptic species, as they are both well documented North Atlantic species. Recently, *P. tenuipes* was recorded from the shelf of the North Bering Sea (Budnikova and Bezrukov 2018), but as in the previous examples, these are likely misidentifications as this species is distributed mainly in the North Atlantic (G.O. Sars 1893; Buhl-Jensen 1986; Lörz et al. 2021). Our present discovery of *Pardalisca magdalenae* sp. nov. at 4185–4182 m extends the depth distribution of the genus *Pardalisca* in the Pacific Ocean to abyssal depths and is the deepest record known. The Pacific citations from Karaman (1974) were all in general proximity to land masses or islands, but not from the open Pacific. With the continued use of molecular techniques and morphological study (integrative taxonomy), it is likely that many more cryptic species of *Pardalisca* will be discovered in the abyssal Pacific.

There are 20 publicly available COI sequences of Amphipoda longer than 500 bp originating from individuals identified as *Pardalisca* (GenBank and BOLD) (Suppl. material 1). They are assigned to *P. endeavouri* (seven sequences, Murdock et al. 2021), *P. tenuipes* (eight sequences, not assigned to any publication), *P. abyssi* (two sequences, not assigned to any publication), *P. cuspidata* (one sequence, not assigned to any publication) and to *Pardalisca* sp. BOLD:ADF7323 (one sequence, Jażdżewska and Mamos 2019). The *p*-distance between the sequence of *P. magdalenae* sp. nov. and the other species varies from 0.22 to 0.27 (Suppl. material 1), which is much higher than the threshold used for amphipod species delimitation (e.g. Tempestini et al. 2018). Important to note is that North Atlantic *Pardalisca* species require revision because individuals morphologically identified as *P. tenuipes* after barcoding appear to be spread between three lineages (Suppl. material 1). Furthermore, the sequence of *P. cuspidata* groups with *P. tenuipes*; this evidence suggests a lack of robust, morphological characters separating these two species, leading to misidentification.

## Supplementary Material

XML Treatment for Pardalisca

XML Treatment for Pardalisca magdalenae

## Additional information

### Author ORCIDs

Karolina Biniek https://orcid.org/0009-0007-2984-9322

Ed A. Hendrycks https://orcid.org/0000-0001-6199-7121

Anna M. Jażdżewska https://orcid.org/0000-0003-2529-0641

### Data availability

All of the data that support the findings of this study are available in the main text or Supplementary Information.

## Supplementary materials

Supplementary material 1 Molecular comparison of *Pardalisca magdalenae* sp. nov. with publicly available sequences of individuals identified as *Pardalisca* Data type pdf https://binary.pensoft.net/file/1568759This dataset is made available under the Open Database License (http://opendatacommons.org/licenses/odbl/1.0/). The Open Database License (ODbL) is a license agreement intended to allow users to freely share, modify, and use this Dataset while maintaining this same freedom for others, provided that the original source and author(s) are credited. Karolina Biniek, Ed A. Hendrycks, Anna M. Jażdżewska
