# Supplementary material for: A new species of Pardalisca Krøyer, 1842 (Crustacea, Amphipoda, Pardaliscidae) from the Clarion-Clipperton Zone in the abyssal central east Pacific
Source: Zookeys. 2026 Mar 24;1274:215–28. doi: 10.3897/zookeys.1274.140692 (PMC13040256; doi:10.3897/zookeys.1274.140692)

Biniek K, Hendrycks EA, Jażdżewska AM (2025) A new species of *Pardalisca* (Crustacea, Amphipoda, Pardaliscidae) from the Clarion-Clipperton Zone in the abyssal central east Pacific. ZooKeys

Supplementary material S1. Molecular comparison of *Pardalisca magdalenae* sp. nov. with publicly available sequences of individuals identified as *Pardalisca*.

Table S1. Details of the sequences available in GenBank and Barcode of Life Datasystems (BOLD) with identification, Barcode Index Number (BIN), accession numbers and source information.

GenBank or BOLD accession number*	Identification	BIN	Source
AMPNB063-13	*Pardalisca abyssi*	BOLD:ACF7223	BOLD, not assigned to publication
AMPNB179-14	*Pardalisca abyssi*	BOLD:ACF7223	BOLD, not assigned to publication
AMPNB064-13	*Pardalisca tenuipes*	BOLD:ACF7223	BOLD, not assigned to publication
AMPNB130-14	*Pardalisca tenuipes*	BOLD:ACM2065	BOLD, not assigned to publication
AMPNB382-16	*Pardalisca tenuipes*	BOLD:ACM2065	BOLD, not assigned to publication
AMPNB465-16	*Pardalisca cuspidata*	BOLD:ACM2065	BOLD, not assigned to publication
HABFA2015-22	*Pardalisca tenuipes*	BOLD:ACM2065	BOLD, not assigned to publication
HABFA2016-22	*Pardalisca tenuipes*	BOLD:ACM2065	BOLD, not assigned to publication
AMPNB445-16	*Pardalisca tenuipes*	BOLD:ACM2065	BOLD, not assigned to publication
MN346574.1	*Pardalisca* sp.	BOLD:ADF7323	Jażdżewska and Mamos 2019
HCHAR419-19	*Pardalisca tenuipes*	BOLD:ACA9853	BOLD, not assigned to publication
MG318376.1	*Pardalisca tenuipes*	BOLD:ACA9853	BOLD, not assigned to publication
PQ734367	*Pardalisca magdalenae* sp. nov.	BOLD:AFU4686	present study
MZ197642.1	*Pardalisca endeavouri*	BOLD:ADM5605	Murdock et al. 2021
MZ197643.1	*Pardalisca endeavouri*	BOLD:ADM5605	Murdock et al. 2021
MZ197647.1	*Pardalisca endeavouri*	BOLD:ADM5605	Murdock et al. 2021
MZ197644.1	*Pardalisca endeavouri*	BOLD:ADM5605	Murdock et al. 2021
MZ197646.1	*Pardalisca endeavouri*	BOLD:ADM5605	Murdock et al. 2021
MZ197648.1	*Pardalisca endeavouri*	BOLD:ADM5605	Murdock et al. 2021
MZ197649.1	*Pardalisca endeavouri*	BOLD:ADM5605	Murdock et al. 2021

* BOLD accession number provided in cases where sequence is not deposited in GenBank.

Jażdżewska AM, Mamos T (2019) High species richness of Northwest Pacific deep-sea amphipods revealed through DNA barcoding. Progress in Oceanography 178: 102184. https://doi.org/10.1016/j.pocean.2019.102184

Murdock SA, Tunnicliffe V, Boschen-Rose RE, Juniper SK (2021) Emergent "core communities" of microbes, meiofauna and macrofauna at hydrothermal vents. ISME communications, 1(1): 27. https://doi.org/10.1038/s43705-021-00031-1

Table S2. COI mean interspecies *p*-distances based on sequences between *Pardalisca* species. Species presented with names and assigned BINs.

	Pardalisca abyssi/tenuipes (ACF7223)	*Pardalisca tenuipes/cuspidata* BOLD:ACM2065)	*Pardalisca* sp. (BOLD:ADF7323)	*Pardalisca tenuipes* (BOLD:ACA9853)	*Pardalisca magdalenae* sp. nov. (BOLD:AFU4686)	*Pardalisca endeavouri* (BOLD:ADM5605)
Pardalisca abyssi/tenuipes (BOLD:ACF7223)						
Pardalisca tenuipes/cuspidata BOLD:ACM2065)	0.24					
Pardalisca sp. (BOLD:ADF7323)	0.26	0.19				
Pardalisca tenuipes (BOLD:ACA9853)	0.21	0.20	0.21			
Pardalisca magdalenae sp. nov. (BOLD:AFU4686)	0.22	0.22	0.23	0.22		
Pardalisca endeavouri (BOLD:ADM5605)	0.24	0.31	0.31	0.29	0.27	

Figure S1. Neighbor-joining tree of *p*-distances of the *COI* sequences of *Pardalisca*. Bootstrap 1000 replicates, only values over 95 shown. Note it is not a phylogenetic tree.

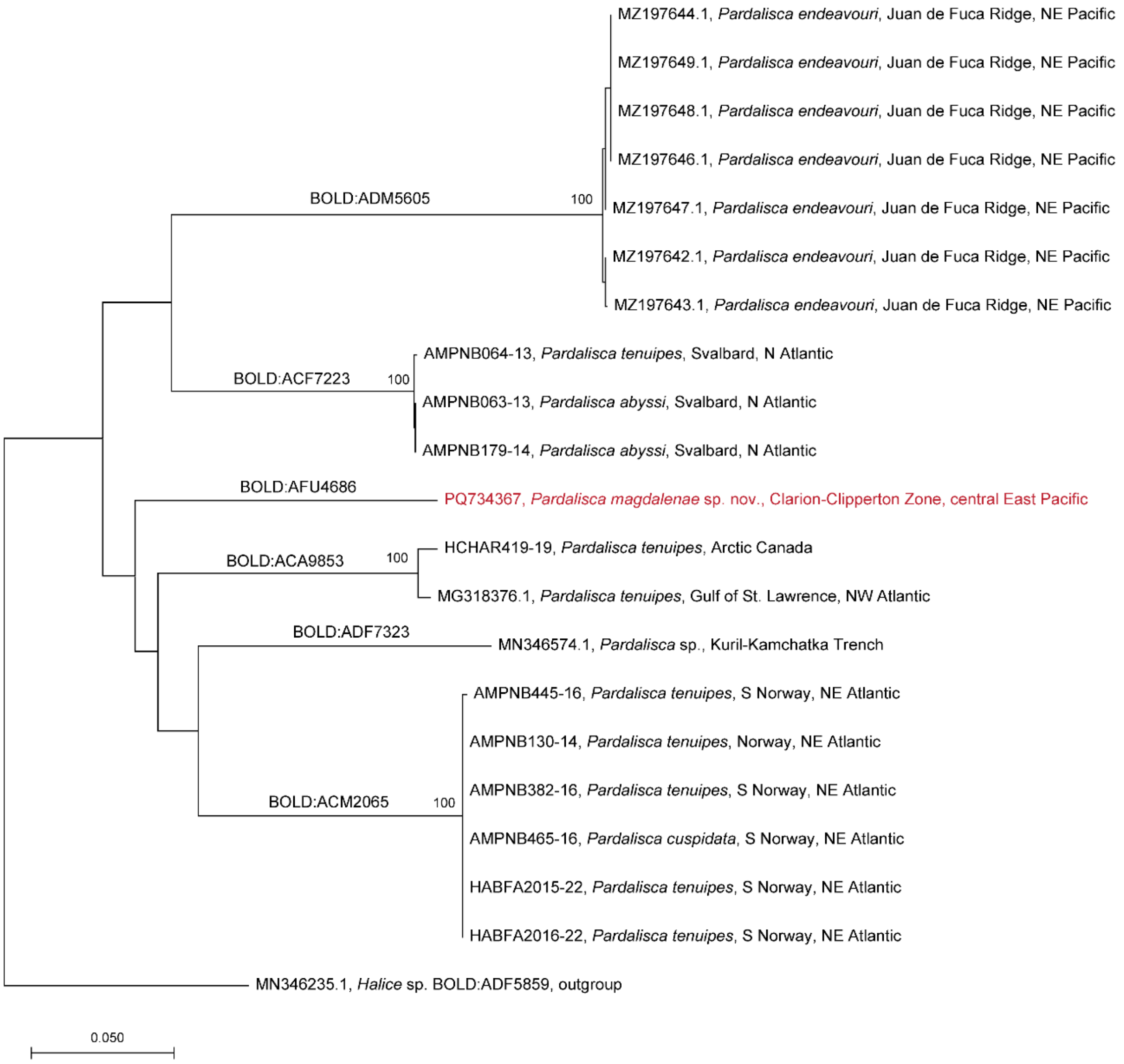

Supplement: Supplementary material 1 — Molecular comparison of Pardalisca magdalenae sp. nov. with publicly available sequences of individuals identified as Pardalisca [file zookeys-1274-215_article-140692__-s001.pdf]
